# Effects of nitrate supplements on cardiopulmonary fitness at high altitude: A meta-analysis of nine randomized controlled trials

**DOI:** 10.1371/journal.pone.0319667

**Published:** 2025-04-09

**Authors:** Chao Kang, Ning Lin, Yanle Xiong, Yi Yang, Jiaojiao Shi, Kaihong Zeng, Xin Ma

**Affiliations:** 1 Department of Clinical Nutrition, The General Hospital of Western Theater Command, Chengdu, Sichuan, China; 2 Li Ka Shing Faculty of Medicine, The University of Hong Kong, Hong Kong SAR, China; 3 Department of Rehabilitation Medicine, The General Hospital of Western Theater Command, Chengdu, Sichuan, China; 4 Department of Health Management Center & Institute of Health Management, Sichuan Provincial People’s Hospital, University of Electronic Science and Technology of China, Chengdu, Sichuan, China; Portugal Football School, Portuguese Football Federation, PORTUGAL

## Abstract

**Background:**

Nitrate is a dietary intervention commonly used to enhance exercise capacity, including cardiopulmonary fitness, yet its effectiveness has been recently questioned at high altitudes. This meta-analysis systematically evaluates the effects of dietary nitrate supplements on cardiopulmonary fitness at high altitude, as reflected in the biomarker of cardiopulmonary fitness, paving the way for informed dietary strategies.

**Methods:**

We conducted a systematic assessment and meta-analysis of randomized controlled trials to examine the effects of dietary nitrate supplementation on biomarkers of cardiorespiratory health at high altitude. Studies were included if they involved healthy individuals (≥16 years of age) engaging in endurance activities such as hiking, long-distance running, mountain climbing, or bicycling at high altitude. Outcomes of interest included nitrite levels (NO_2_-), maximal oxygen uptake (VO_2max_), heart rate (HR), perceived exertion (RPE), and pulse oxygen saturation (SpO_2_). Exclusion criteria included duplicate publications, non-human studies, studies with missing data that could not be retrieved, non-randomized clinical trials, and non-original research articles such as conference papers, expert consensus, or reviews. Our search for articles was conducted across PubMed, Scopus, Web of Science, and Embase, without any language restrictions. A random effects model was employed for quantitative data analysis, utilizing Standardized Mean Difference (SMD) and 95% confidence intervals as summary statistics. The methods and results were reported according to the PRISMA2020 statement.

**Results:**

A total of 9 studies with a sample size of 161 cases were included in the analysis. The meta-analysis indicated that dietary nitrate supplement significantly elevated NO_2_^-^ concentration (95% CI: 1.38 to 3.12; SMD = 2.25, *P* < 0.00001; *I*^*2*^ = 70%). However, there was no significant effect observed on VO_2max_ (95% CI: -0.58 to 0.23; SMD = -0.17, *P* = 0.76; *I*^*2*^ = 0%), HR (95% CI: -0.31 to 0.23; SMD = -0.04, *P* = 0.77; *I*^*2*^ = 0%), RPE scores (95% CI: -0.49 to 0.18; SMD = -0.16, P = 0.36; *I*^*2*^ = 0%), and SpO_2_ percentage (95% CI: -0.36 to 0.20; SMD = -0.08, *P* = 0.58; *I*^*2*^ = 0%).

**Conclusions:**

The current meta-analysis indicates that dietary nitrate intake is less correlated with cardiopulmonary fitness at high altitudes, and further research is required to clarify its impact on exercise capacity.

## 1. Introduction

The unique climatic characteristics of high-altitude environments are characterized by low atmospheric pressure, partial pressure of oxygen, and temperature, along with enhanced solar ultraviolet radiation [1]. As individuals accustomed to lower altitudes ascend rapidly, their bodies undergo physiological adjustments, including an increase in red blood cell count and a decrease in hemoglobin levels, alterations in respiratory and pulmonary ventilation, and changes in the hematological system (e.g., SpO_2_.) [2]. These adjustments typically occur gradually as the body needs time to acclimate to the high-altitude environment. Moreover, the high-altitude environment poses challenges for sports enthusiasts, as hypoxia disrupts homeostasis, impairs respiratory center function, leads to increased fatigue, and significantly diminishes physiological function and athletic performance [3,4]. In the 21st century, however, altitude training has emerged as a standard training regimen for many athletes, enhancing muscle buffering capacity and myoglobin concentration, thereby improving endurance performance and other athletic abilities at sea level [5]. Consequently, there is an urgent need to develop effective strategies to enhance cardiopulmonary fitness at high altitudes, thereby improving the quality of life and exercise capacity of individuals from lower altitudes when they ascend, as well as boosting the exercise capacity of athletes training in high-altitude environments. Increasing dietary nitrate intake is a complementary strategy with the potential to further enhance the benefits gained from high-altitude training, specifically in improving exercise capacity and cardiopulmonary fitness.

Nitrate is a naturally occurring anion in the human body, and NO_3_- can be converted to NO_2_- by enteric bacteria in the mouth and gut [6]. Subsequently, under conditions of low oxygen tension and weakly acidic pH, NO_2_- can be converted to nitric oxide (NO) by enzymes such as deoxyhemoglobin and xanthine oxidase [7]. NO has been shown to play a potentially beneficial role in several physiological processes related to exercise capacity, including improvements in cardiopulmonary fitness, endurance, and muscle strength [8,9].

There is an increasing interest in the potential benefits of nitrate for individuals residing at high altitudes, particularly for those entering such environments for the first time. However, the findings from previous studies have been inconsistent [10]. The aim of this study is to systematically analyze and evaluate the impact of nitrate supplementation on the exercise capacity at high altitudes, as reflected in the biomarker of cardiopulmonary fitness, thereby offering a foundation for the rational implementation of nutritional interventions. To achieve this, an extensive literature search was conducted across preprint platforms and databases, with the goal of encompassing all pertinent trials to uncover knowledge gaps and support decision-making processes.

## 2. Methods

### 2.1. Search strategy

This systematic review was conducted in accordance with the guidelines outlined in the Cochrane Handbook for Systematic Reviews and the Preferred Reporting Items for Systematic Reviews and Meta-Analyses (PRISMA) statement [11]. The Meta analysis was not registered before. Potential studies were identified through systematic searches of online databases, including PubMed (Medline), Embase, and Web of Science-Science Citation Index, as well as others, to identify potential studies on June 2024. The searches were conducted from the inception of each database, with no restrictions on language or year of publication. Search terms used to identify potential studies included (“nitrate” OR “nitrite” OR “beetroot”), (“high altitude” OR “plateau” OR “mountain” OR “hypoxia” OR “anoxia”), and (“sport” OR “exercise” OR “training” OR “performance” OR “strength” OR “resistance”). Validated filters were used to identify randomized controlled trials. Details of the PubMed search terms are provided in S1 Table. Additionally, a manual search was conducted on preprint platforms (medRxiv and Research Square) and other databases (CINAHL, China National Knowledge Internet databases, Wanfang Database, and Scielo). For research published in languages other than English, we either use translation tools or seek the help of colleagues who are fluent in those languages to accurately interpret the content. To supplement our searches of academic databases, we also conducted searches in gray literature sources, including ProQuest Dissertation and Thesis, Ethos, and Clinical Trials.

### 2.2. Eligibility criteria

We included studies in which subjects met the following criteria: (1) Participants must be healthy individuals (over the age of 16) engaged in endurance activities at high altitudes, including trekking, distance running, climbing, or cycling. (2) studies provided sufficient details in the report, including study design, participant characteristics, intervention details, outcome measures, and results necessary for data extraction and analysis. (3) Outcomes of interest included nitrite levels (NO_2_-) for evaluating the efficacy of dietary nitrate supplementation, parameters of cardiopulmonary fitness such as maximal oxygen uptake (VO_2max_), heart rate (HR) and rating of perceived exertion (RPE), and pulse oxygen saturation (SpO_2_) for high-altitude adaptation. We excluded (1) duplicate literature, (2) studies using cells or animals, (3) literature with missing data that could not be retrieved from the authors, (4) not randomized clinical trials (RCTs), and (5) articles from other study types, such as conference papers, expert consensus, and reviews.

### 2.3. Screening and data extraction

A standardized data collection form was designed and developed in Microsoft Excel. Two independent reviewers (Y.X. and Y.Y.) screened studies retrieved from electronic databases to identify relevant texts. Irrelevant titles were excluded, and the remaining articles were systematically screened for eligibility through their abstracts and full texts. Data were extracted from relevant research papers. If information/data was missing, the corresponding author was contacted via email to request the missing data, or the study was excluded if the required information could not be obtained from the authors. The information extracted included the title, authors, year, country, simulated altitude, type of exercise, number of subjects, age, gender, type and dose of nitrates, duration of intervention, outcome indicators for subjects, NO_2_^-^ levels, VO_2max_(or VO_2_ peak), RPE, HR, and SpO_2_.

### 2.4. Quality assessment

The risk of bias for the included studies was independently assessed by two investigators (YX and YY), with cross-validation of the results. Quality assessment was conducted using the Cochrane Risk of Bias tool 2.0 (ROB2) [12]. Information on the risk of bias was extracted and integrated into the study characteristics within Review Manager (version 5.4). The tool encompasses the following elements: randomized sequence generation (selective bias), allocation concealment (selective bias), blinding of participants and personnel (implementation bias), blinding of outcome assessors (measurement bias), incomplete outcome data (follow-up bias), selective reporting of outcomes (reporting bias), and other biases. Disagreements over the rating of a study were resolved by having all authors review the article and reach a consensus. The included trials were categorized into “low risk of bias,” “unclear risk of bias,” or “high risk of bias.”

### 2.5. Statistical analysis

Meta-analysis of the included studies was performed using Review Manager v.5.4 (The Cochrane Collaboration, Copenhagen, Denmark). and STATA v.17.0 software (College Station, TX: StataCorp LLC). Effect sizes for continuous variables were expressed as standardized mean difference (SMD) and 95% confidence intervals (CI) were analyzed as summary statistics. To achieve comparability across studies, Data presented as medians and interquartile ranges (IQR) were converted to means and standard deviations (SD) using Hozo’s method [13]. For variables with the standard errors (SE) reported, SD was calculated by the following equation: SE ×  √  (sample size). The post-intervention outcome indicators (NO_2_^-^ levels, VO_2max_, RPE, HR, and SpO_2_) or the changes from baseline (or at sea levels) were selected for further analysis (See more details in Table 1). The DerSimonian and Laird method was employed to estimate the between-study variance. Random-effects models were used based on heterogeneity of study outcomes. Inter-study heterogeneity was assessed using a chi-squared test and the *I*^*2*^ statistic; if *I²* ≥  50% and *P*-value <  0.1, the outcome was considered significantly heterogeneous. Subgroup analyses based on gender was also performed. Publication bias was assessed by funnel plots, Begg’s rank correlation and Egger’s weighted regression test. If there was evidence of funnel plot asymmetry, the ‘trim and fill’ method was used to estimate potentially missing studies.

**Table 1:** Characteristics of the studies included in this review.

Author, Year	Country	Population	Sample size (n)	Age (Years)	Altitude (m)	Exposure state	Sports mode	Research method	Intervention Regimen	Intervention dose	Control Regimen	Duration	Outcome indicator
Arnold, 2015 [21]	UK	Well-trained competitive male runners	10	37 ± 13	2500	Intermittent exposure at simulated altitude	Running (10 km treadmill time trial and an incremental exercise test)	Double-blind repeated measures crossover design	Concentrated beetroot juice	70mL (7mmol nitrates)	Same texture, taste and appearance with negligible nitrate concentration placebo	Studying period: 17 days (Nitrate supplementation: 3 days)	①②③④⑤; ① was recorded before and 2.5 h after the ingestion of beetroot juice or placebo at each visit to the laboratory. The later data was used for analysis. ② was assessed using a continuous incremental exercise test on a motorized treadmill and was determined as the highest 30-s average at any given time point. The change between 4,000m altitude and sea level were used for analysis. ③④⑤ were recorded during the final 15 s of each incremental stage at 4,000 m altitude. And these data at 100% VO_2max_ were used for analysis.
Hennis, 2016 [22]	UK	Healthy male students	40	16 ± 1	5300	Continuous altitude exposure	Climbing (2-minute step test)	Single-blind parallel group randomized controlled design	Concentrated beetroot juice	140 mL (10mmol nitrates)	Concentrated blackcurrant juice	Studying period: 11days (Nitrate supplementation: 7 days)	③④; ③④ were measured at rest (after a 5-min stabilization period) and in the last 10 seconds of a 2-min stepping exercise each morning. Data collected from the post-exercise measurement on the 11^th^ day were used for analysis.
Shannon, 2016 [23]	UK	Healthy males	12	24.4 ± 4.3	2500	Intermittent exposure at simulated altitude	Running (2 × 15 minute bouts of steady-state running, 1500m time trial)	Randomized double-blind placebo-controlled design	Concentrated beetroot juice	138ml (15.2 mmol nitrates)	Placebo with negligible nitrates	Studying period: Five occasions within a six week period. (Nitrate supplementation: The same day of one occasions [4th or 5th].)	①②③④⑤; ① was measured pre-supplementation, 30, 60, 90, 120, 150 (pre-hypoxic exposure) and 180 (pre-exercise) minutes post-supplementation, following each bout of steady-state exercise, and immediately post-TT. Data of 1h post supplementation was used for analysis. ② was measured by incremental running tests to volitional exhaustion in normoxia and at a simulated altitude of 2,500m. The data of 45% VO_2max_ and 65% VO_2max_ at 2,500m were used for analysis. ③④ were measured pre-hypoxic exposure, pre-exercise, during the final 2 min of each steady-state exercise bout, and immediately post-TT. The value at post-TT was used for analysis. ⑤was measured during the final 2 min of each steady-state exercise bout and immediately post-TT. The value at post-TT was used for analysis.
Rossetti, 2017 [24]	UK	Recreationally-active males	20	22 ± 4	4219	Intermittent exposure at simulated altitude	Weighted trekking (Loaded walking whilst carrying a 15 kg rucksack on a motorized treadmill)	Randomized-double-blinded placebo-controlled crossover design.	Concentrated beetroot juice	70ml (6.4 mmol nitrates)	Placebo with negligible nitrates	Studying period: About 22 days (Nitrate supplementation: 6 days)	①③④⑤; ① were measured immediately before (day 0), on day 5 (hypoxia) and after day 6 (pre-hypoxia) of each supplementation phase. Data from day 5 were used for analyses. ③④⑤ were recorded at 2-minute intervals during Submaximal exercise responses and recorded every minute during TTE exercise testing.These value at 100% isotime during the Time to Exhaustion (TTE) test was used for analysis.
Shannon, 2017 [25]	UK	Healthy males	10	23 ± 3	4300	Intermittent exposure at simulated altitude	Running (45 min walk and 3 km time-trial)	Randomized controlled design	Concentrated beetroot juice	140ml (12.5 mmol nitrates)	Placebo with negligible nitrate concentration	Studying period: Five separate occasions within a 7 week period (Nitrate supplementation: The same day of two occasions at 3,000 m and 4,300m.)	①③④⑤; ① was recorded after a 5-minute break in a seated position. The value of 4300 m for simulated altitude exposure was used for the analysis. ③④ were measured after blood pressure was measured at sitting rest, pre-exercise hypoxic rest, during the last 5 minutes of steady-state walking, immediately after TT, and before leaving the hypoxic chamber. These value of 4300 m for simulated altitude exposure at post-TT ware used for analysis. ⑤ was recorded after the final 5 min steady-state exercise and immediately post-TT. The value of 4300 m for simulated altitude exposure at post-TT ware used for analysis.
Kent, 2019 [26]	Australia	Moderately-trained, male	12	22.3 ± 2.6	3000	Intermittent exposure at simulated altitude	Cycling (front-access bicycle Ergometer, including 3 parts: Warm-up, Main-set and Cool-down)	Double blind, repeated-measures, counter-balanced design	Concentrated beetroot juice	2 × 70ml (6.45 mmol nitrates)	Placebo with negligible nitrates	Studying period: Not mentioned (Nitrate supplementation: 1 day prior to each exercise session)	③④⑤; ③④⑤ were measured after a 10-minute warm-up and the final sprint of each RSE set. The value at RSE4 was used for analysis.
Robinson, 2020 [27]	UK	trained males	8	23 ± 4	2400	Intermittent exposure at simulated altitude	Running (repeated 90 s intervals at 110% of peak treadmill velocity)	Repeated-measures, crossover design	Concentrated beetroot juice	140ml (12.4 mmol nitrates)	Placebo with negligible nitrates	Studying period: 4 weeks (Nitrate supplementation: 7 days)	①④; ① was measured before the test and 5-min recovery after test. The data from the 5-minute recovery period following the post-test at 2,400 meters were used for analysis. ④ was continuously detected before and after the test. The mean value over the final 3 min of wash-in at 2,400 meters were used for analysis.
Marshall 2021 [28]	UK	Healthy adults	22	28 ± 12	4800	Continuous altitude exposure	Climbing (23-day expedition starting from Kathmandu [1400 m] trekking to a maximum altitude of 5755 m [Tepsi-Lapsa Pass])	Single-blinded randomized control study	Concentrated beetroot juice	140ml (12.5 mmol nitrates)	Non-nitrate, same calorie placebo	Studying period: 20 days (Nitrate supplementation: 20 days)	①③④⑤; ① were measured before breakfast ③④ were measured after participants seated at rest for 3–5 min ⑤ was recorded after the evening meal on each expedition day These value of 4800 m for altitude exposure ware used for analysis.
Hennis, 2022 [29]	UK	healthy volunteers	27	21 males: 28.9 ± 5.2	4559	Continuous altitude exposure	Cycling (Two test: 1.Submaximal constant work rate test; 2.Maximal exercise test)	Randomized, double blind, placebo-controlled factorial design	Concentrated beetroot juice	0.18 mmol/kg/day nitrates	Placebo with negligible nitrates	Studying period: Not mentioned (Nitrate supplementation: 4days at sea level; 8 days at high altitude)	①②③; A symptom-limited, incremental ramp cycling protocol to volitional exhaustion was performed at SL and HA by each participant to determine ② ③ was collected on mornings at sea level and on the 1st, 3rd and 5th mornings of the study period at high altitude, at the 5 time points of the submaximal constant work rate exercise tests; at the beginning of the test,2 minutes before the end of the 20 W, 40 W and 60 W stages and immediately before finishing the test during unloaded recovery These value of high-altitude exposure (linear mixed modeling) ware used for analysis

Note: ① NO_2_^ −^ levels; ② VO_2max_; ③ HR_;_ ④ SpO_2_ ⑤ RPE.

## 3. Results

### 3.1. Search results

**Fig 1:**
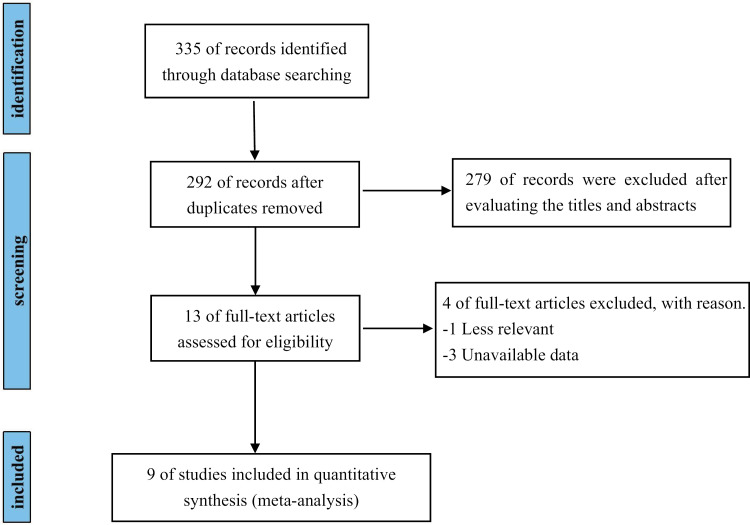
Flow diagram for identification of studies in the systematic review.

A total of 335 papers were identified through the combined database search, with 43 duplicates excluded. Following an assessment of the titles and abstracts, 279 papers were excluded, leaving 13 articles for full-text screening. One study was excluded because it focused on the effects of nitrate supplementation on non-cardiopulmonary outcomes at high altitude, which were not relevant to the meta-analysis's objective, and an additional 3 articles were excluded due to the absence of necessary outcome measures. Consequently, nine studies of RCTs were included, comprising a total sample of 161 cases (Fig 1 and S2 Table).

### 3.2. Study characteristics

This analysis encompasses nine studies published between 1985 and 2022, conducted in two countries: the UK and Australia. Participants were randomly allocated to control and intervention groups to mitigate allocation bias. The mean ages of the subjects in the included studies varied from 16 to 37 years. All studies employed nitrates (beetroot juice) as the intervention, with the control group receiving a placebo corresponding to the intervention. The study population comprised physically active or trained healthy individuals (free from injuries, diseases, prescription drugs, illicit social drugs, tobacco, and other nutritional supplements), including young adult males and females. The duration of the interventions ranged from 11 days to 7 weeks, and the specified period of actual consumption of the nitrate supplement was also included (Table 1).

### 3.3. Subject characteristics

A total of 161 unique participants were included in the meta-analysis. Due to the crossover design of some studies, these participants contributed data to both intervention and control phases, yielding 224 data points, with 50.45% assigned to the intervention (nitrate) group and 49.56% to the control group. The gender composition of the subjects was predominantly male, with 77.88% and 77.48% of the participants in the intervention and control groups, respectively. Similarly, individuals > 18 years old constituted the primary subject population for the trial, comprising 81.42% and 88.88% of the intervention and control groups, respectively. Most studies focused on areas with altitudes greater than 3500m, with 67.26% and 66.67% of the participants in the treatment and control groups, respectively. Among the different study regions, the UK represented 92.5% of the participants in both the intervention and control groups. Running accounted for the largest proportion of exercise patterns across studies, comprising 30.97% and 31.53% of the participants in the treatment and control groups, respectively (Table 2).

**Table 2:** Demographic characteristics of participants.

Characteristics	Intervention	Placebo
**Total numbers, n (% total)**	113(50.45%)	111(49.56%)
**Gender**		
Males	88(77.88%)	86(77.48%)
Males + Females	25(22.12%)	25(22.52%)
**Age**		
Adults	92(81.42%)	92(88.88%)
Minors	21(18.58%)	19(17.12%)
**Altitude**		
<3500m	37(32.74%)	37(33.33%)
>3500m	76(67.26%)	74(66.67%)
**Nation**		
UK	111(98.23%)	99(89.19%)
Australia	12(1.77%)	12(10.81%)
**Sport modes**		
Running	35(30.97%)	35(31.53%)
Cycling	26(23%)	26(23.42%)
Climbing	32(28.32%)	30(27.03%)
Weighted trekking	20(17.70%)	20(18.02%)

### 3.4. Risk of bias

Three of the nine included studies were found to have a high risk of bias overall (Hennis, 2016 & 2022; Marshall, 2021), while three had a low risk of bias overall (Arnold, 2015; Rossetti, 2017 and Shannon, 2016). Furthermore, one study had issues with random sequence generation (Kent, 2019), three had problems with allocation concealment (Robinson, 2020; Hennis, 2022; Kent, 2019), four had issues with the blinding of outcome assessment (Robinson, 2020; Kent, 2019; Marshall 2021; Shannon, 2017), and one had issues with incomplete outcome data (Hennis, 2022, Fig 2 and S3 Table).

**Fig 2:**
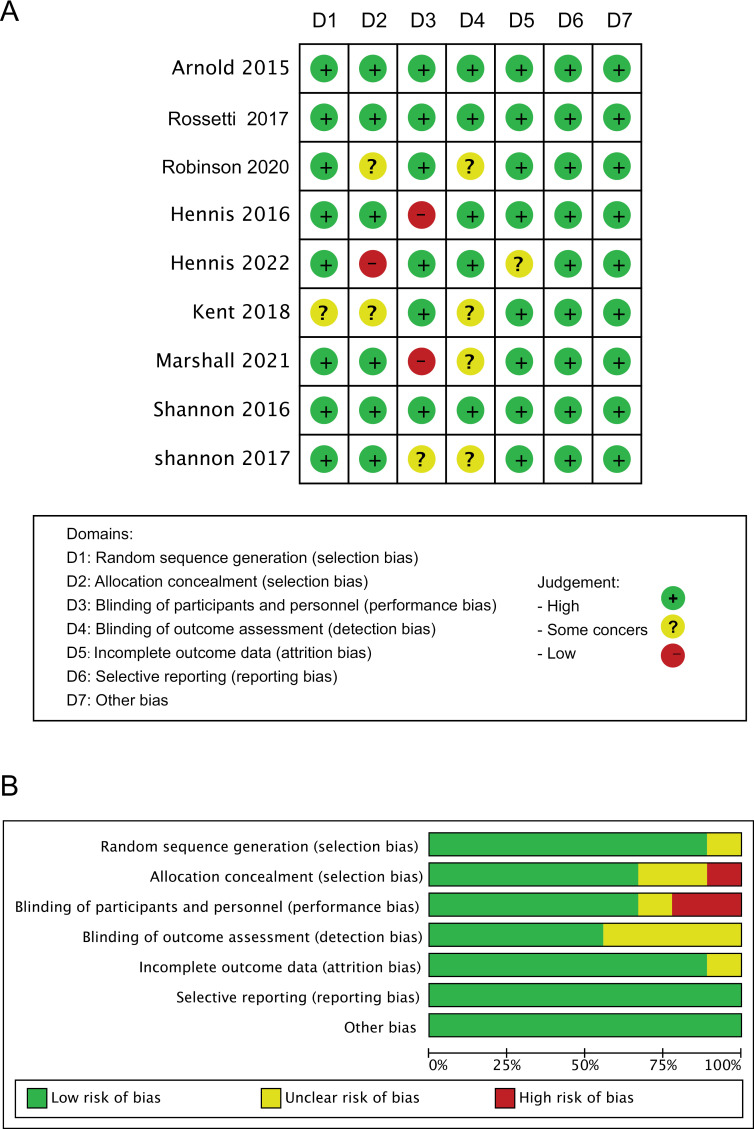
Risk of bias (ROB) analysis highlighting results in all domains examined within the nine identified studies (A) and overall risk of bias for included studies (B).

### 3.5. Parameters of cardiopulmonary fitness

**Fig 3:**
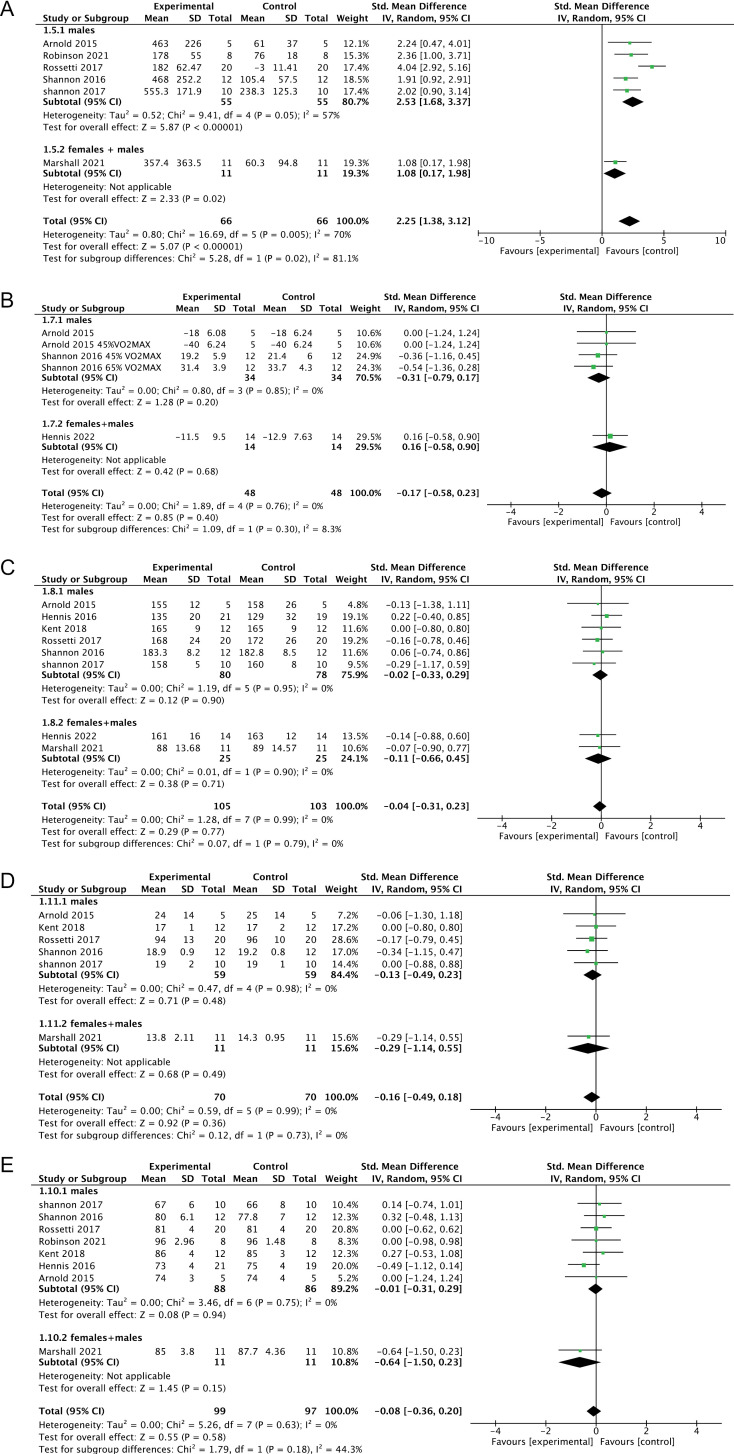
Forest plot displaying weighted standardized mean difference and 95% confidence intervals for the effect of nitrate intervention on NO_2_^-^ levels (A), VO_2max_ (B), HR (C), RPE (D) and SpO_2_ (E) levels.

The meta-analysis of nine studies involving nitrate interventions indicated that nitrate ingestion significantly increased NO_2_ ⁻ concentrations (95% CI: 0.7 to 2.33; SMD = 1.52, *P* = 0.004; *I*^*2*^ = 71%, Fig 3A). Further subgroup analyses stratified by gender demonstrated significant heterogeneity in the meta-analysis outcomes between studies involving subjects of different genders. This suggests that the gender composition of the subjects may contribute to the observed heterogeneity in the meta-analysis of NO_2_^-^ levels (Males: 95% CI: 1.68 to 3.37, SMD = 2.53, *P* = 0.05, *I*^*2*^ = 57%, Fig 3A). Additionally, the meta-analysis showed that nitrate interventions had no significant effect on VO_2max_ (95% CI: -0.58 to 0.23; SMD = -0.17, *P* = 0.76; *I*^*2*^ = 0%, Fig 3B), HR (95% CI: -0.31 to 0.23; SMD = -0.04, *P* = 0.77; *I*^*2*^ = 0%, Fig 3C), RPE scores (95% CI: -0.49 to 0.18; SMD = -0.16, P = 0.36; *I*^*2*^ = 0%, Fig 3D), and SpO_2_ percentage (95% CI: -0.36 to 0.20; SMD = -0.08, *P* = 0.58; *I*^*2*^ = 0%, Fig 3E). Subgroup analyses of the above outcome indicators by gender showed that the gender composition of the subjects did not have a significant effect on these indicators.

### 3.6. Publication bias

**Fig 4:**
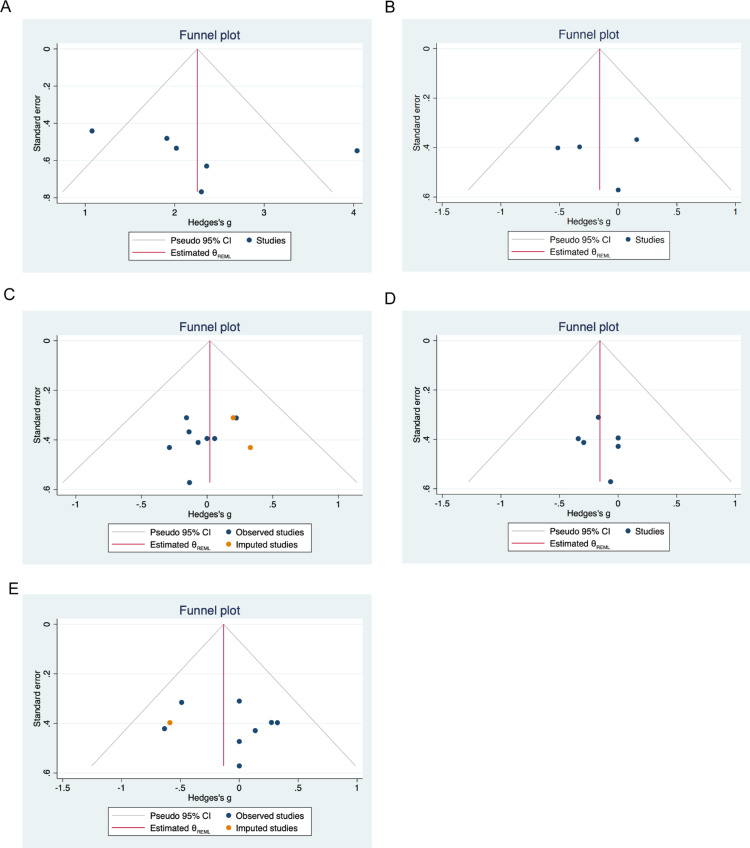
Funnel plot detailing publication bias in the studies reporting the impact of dietary nitrate on NO_2_^-^ levels (A), VO_2max_ (B), HR (C), RPE (D) and SpO_2_ (E) levels.

The visual examination of funnel plots indicated asymmetries in the meta-analysis of NO_2_^-^ levels, VO_2max_, HR, RPE and SpO_2_ levels. Following the trim-and-fill correction, zero, zero, two, zero and one studies were potentially imputed for the analyses of NO_2_^-^ levels, VO_2max_, HR, RPE, and SpO2, respectively. This indicates that there was no evidence of publication bias in our meta-analysis of nitrate. These findings are illustrated in Fig 4. The outcomes of the two Egger’s regression tests and Begg’s rank correlation analysis are summarized in Table 3. The results of sensitivity analysis demonstrated that the pooled effects of dietary nitrate on NO_2_^-^ and above biomarker of cardiopulmonary fitness were not changed after imputation using a correlation coefficient of 0.5.

**Table 3:** Imputed effect sizes and the results of Begg’s rank correlation and Egger’s regression tests for the meta-analysis on parameters of thyroid function. ^a^The number of imputed studies according to the trim and fill correction method; ^b^Begg’s rank correlation test; ^c^Egge’s weighted regression test.

	n^a^	SMD	95% CI	*P-value* ^b^	*P-value* ^c^
NO_2_^-^	0	4.220	-8.781 to 14.653	0.133	0.525
VO_2max_	0	2.479	-7.282 to 8.497	1.000	0.822
HR	2	2.072	-5.965 to 4.173	0.711	0.681
RPE	0	2.376	-6.192 to 6.999	1.000	0.873
SpO_2_	1	1.932	-3.620 to 5.833	0.902	0.588

## 4. Discussion

The objective of this meta-analysis was to examine the impact of nitrate interventions on exercise capacity at high altitude. The results demonstrated that nitrate ingestion increased NO_2_^-^ levels, yet did not significantly influence VO_2max_, HR, RPE points and SpO_2_ percentage. These findings suggest that while nitrate supplementation may enhance certain physiological markers (e.g., NO₂^-^ levels), it does not necessarily translate into improved overall exercise capacity or cardiopulmonary fitness in high-altitude.

Exercise at high altitude poses life-threatening risks. As a result, a number of studies are being conducted to explore specific dietary interventions to improve adaptation to high altitude and to enhance cardiopulmonary fitness and exercise capacity [14]. In recent years, nitrates have garnered significant attention for their potential as performance enhancers in flatland regions. According to the latest consensus statement from the International Olympic Committee (IOC), nitrates are recognized as one of the few dietary supplements with a direct positive effect on athletic performance [15]. To our knowledge, there is a limited number of RCTs investigating the effects of dietary nitrates in high-altitude environments. This study represents the first meta-analysis to evaluate the impact of dietary nitrate supplementation on exercise capacity and cardiopulmonary fitness specifically in high-altitude conditions. The results of the meta-analysis indicated that nitrate intervention significantly increased NO2^-^ concentrations in subjects. NO₂ ⁻ is known to serve as a reservoir for the synthesis of NO precursors. Under conditions of hypoxia, NO reduces both oxygen consumption and adenosine triphosphate (ATP) consumption during exercise, mediating smooth muscle relaxation, promoting vasodilation, and increasing oxygen delivery to skeletal muscle. Subsequent improvements in the function and efficiency of type II muscle fibres in skeletal muscle were associated with positive effects of dietary nitrate on cardiopulmonary fitness [15,16]. While nitrate supplementation demonstrated a modest enhancement in cardiopulmonary fitness at sea level, further research is needed to fully elucidate its impact on exercise capacity at high altitude. Furthermore, significant heterogeneity was observed in the meta-analysis results of NO₂ ⁻ interventions between genders. This may be due to the small sample of female participants or indicate a true gender difference in response. An RCT showed that acute nitrate supplementation significantly lowered blood pressure in males, but not in females. Given NO₂ ⁻ ‘s vasodilatory and blood pressure-lowering effects, the more pronounced response in males suggests that gender plays a key role in modulating the physiological response to nitrate. Additionally, since the menstrual cycle affects cardiovascular control in women, further research is needed to investigate nitrate responses across different female populations, including postmenopausal women [17].

VO_2max_ is the international gold standard for evaluating cardiopulmonary fitness, which reflects the maximum capacity of muscles and lungs to absorb, transport and utilize oxygen during prolonged strenuous exercise [18]. At high altitude, reduced air pressure and lower oxygen levels can result in hypoxia in the blood and lungs. When exercising in this particular environment, the HR increases almost linearly with altitude, which is thought to be a compensatory mechanism for the hypoxic environment, while a lower heart rate indicates an increase in the reserve capacity of the heart and an increase in exercise capacity. SpO_2_ serves as a good indicator of the degree of hypoxia. In hypoxic environments, insufficient ventilation can lead to a significant decrease in SpO_2_, potentially resulting in altitude illnesses [19,20]. RPE is a self-perceived fatigue scale that assesses the intensity of exercise through the self-perception of exertion during physical activity, with higher scores indicating greater exertion. Although no significant effects were observed on other outcome measures, there was a trend towards a decrease in HR and RPE, while VO_2max_ showed a tendency to increase. Previous study demonstrated that short-term nutritional supplementation with nitrate did not affect the oxygen cost, SpO_2_, HR, or RPE under hypoxic conditions at high altitude [21], suggesting a lack of strong evidence to improve performance in trained athletes, especially under such hypoxia condition.

The current meta-analysis possesses several strengths. To the best of our knowledge, it represents the first meta-analysis investigating the impact of dietary nitrate on cardiopulmonary fitness. We conducted a comprehensive and systematic search for relevant studies published between 1987 and 2022, providing a thorough summary of the evidence on the relationship between nitrate intake and performance in endurance sports. However, this study also has certain limitations. For instance, we did not perform separate analyses based on the duration of intervention because most studies focus on short-term interventions. Although the study's duration ranged from 11 days to 7 weeks, the nitrate supplement interventions were generally short-term, with some participants receiving supplements prior to exercise testing or for just the first 3 days. Besides, the analysis was limited by the inclusion of a small number of clinical randomized trials, and variations in the methodologies across studies (such as differences in training status, exercise protocols, supplementation regimens, and environmental conditions) may have influenced the effects of dietary nitrate. Additionally, the majority of the studies involved male participants, which may not accurately reflect the broader demographic composition of the general population. Moreover, sex differences were not thoroughly explored, which could affect the generalizability and reliability of the findings. This Meta-analysis also focused primarily on active individuals with an athletic training background, excluding untrained populations. As a result, the findings may be more applicable to trained or active individuals and may not fully generalize to the broader population. In addition, the age range of subjects was limited and lacked generalisability to all age groups. Additionally, this meta-analysis did not include data on respiratory rate as an outcome measure, primarily due to the limited number of studies that reported this variable. Future research may more comprehensively incorporate respiratory rate as an outcome indicator. Consequently, more rigorous, high-quality intervention studies are required to validate the conclusions drawn from this meta-analysis.

Despite recent advancements in understanding the physiological changes induced by nutrition at high altitude, the potential for optimizing the effects of hypoxic exercise through specific nutritional supplements remains an emerging area of research. Notably, nitrate supplementation does not appear to significantly enhance exercise capacity. This may be due to the limited number of studies exploring nutritional strategies to improve athletic performance in high-altitude environments, as well as variations in research methodologies. The effectiveness of targeted nutritional supplements in enhancing cardiopulmonary fitness and exercise capacity at high altitude remains a subject of ongoing debate and warrants further investigation through larger, more rigorous studies conducted in plateau regions.

## Supporting information

S1 Table Search strategy for included studies. (DOCX)

S2 Table List of all the studies identified from literature search along with the reasons for exclusion. (XLSX)

S3 Table ROB 2 tool for included RCT. (DOCX)

S1 File Data used in meta-analyses. (XLSX)

S2 File PRISMA_2020_checklist. (DOCX)
