# Supplementary material for: Effects of nitrate supplements on cardiopulmonary fitness at high altitude: A meta-analysis of nine randomized controlled trials
Source: PLoS One. 2025 Apr 9;20(4):e0319667. doi: 10.1371/journal.pone.0319667 (PMC11981189; doi:10.1371/journal.pone.0319667)
Supplement: S1 Table — (DOCX) [file pone.0319667.s001.docx]

Supplementary table 1. Search strategy for included studies.

| MEDLINE via PubMed | #1 "nitrate"[All Fields] OR "nitrite"[All Fields] OR "beetroot"[All Fields]  #2 "high altitude"[All Fields] OR "plateau"[All Fields] OR "mountain"[All Fields] OR "hypoxia"[All Fields] OR "anoxia"[All Fields]  #2"sport"[All Fields] OR "exercise"[All Fields] OR "training"[All Fields] OR "performance"[All Fields] OR "strength"[All Fields] OR "resistance"[All Fields]  #1 AND #2 AND #3  **Updated Search**  #3 Limited : clinical trial, and RCT | June 8th  2024 |
| --- | --- | --- |
| Scopus via Elsevier | #1TITLE-ABS-KEY(nitrate) OR TITLE-ABS-KEY(nitrite) OR TITLE-ABS-KEY(beetroot)  #2 TITLE-ABS-KEY(high altitude) OR TITLE-ABS-KEY(plateau) OR TITLE-ABS-KEY(mountain) OR TITLE-ABS-KEY(hypoxia) OR TITLE-ABS-KEYanoxia)  #3 TITLE-ABS-KEY(sport) OR TITLE-ABS-KEY(exercise) OR TITLE-ABS-KEY(training) OR TITLE-ABS-KEY(performance) OR TITLE-ABS-KEY(strength) OR TITLE-ABS-KEY(resistance)  #1 AND #2 AND #3  **Updated Search**  #3 TITLE-ABS-KEY (randomized AND controlled AND trial ) OR TITLE-ABS-KEY (randomized AND clinical AND trial ) OR TITLE-ABS-KEY (clinical AND trial ) OR TITLE-ABS-KEY (RCT) | June 8th  2024 |
| Embase via Elsevier | #1 "nitrate" OR "nitrite" OR "beetroot"  #2 "high altitude" OR "plateau" OR "mountain"OR "hypoxia" OR "anoxia"  #3 "sport" OR "exercise"OR "training" OR "performance" OR "strength" OR "resistance"  #1 AND #2 AND #3  **Updated Search**  #3 - 'crossover procedure':de OR 'double-blind procedure':de OR 'randomized controlled trial':de OR 'single-blind procedure':de OR random*:de,ab,ti OR factorial*:de,ab,ti OR crossover*:de,ab,ti OR ((cross NEXT/1 over*):de,ab,ti) OR placebo*:de,ab,ti OR ((doubl*NEAR/1 blind*):de,ab,ti) OR ((singl* NEAR/1 blind*):de,ab,ti) OR assign*:de,ab,ti OR allocat*:de,ab,ti OR volunteer*:de,ab,ti | June 9th  2024 |
| Web of Science-Science Citation Index and Social Sciences Citation Index via Clarivate | #1 TS=(nitrate) OR TS=(nitrite) OR TS=(beetroot)  #2 TS=(high altitude) OR TS=(plateau) OR TS=(mountain) OR TS=(hypoxia) OR TS=(anoxia)  #3 TS=(sport) OR TS=(exercise) OR TS=(training) OR TS=(performance) OR TS=(strength) OR TS=(resistance)  #1 AND #2 AND #3  **Updated Search**  #3 Limited : TS=(randomized clinical trial) OR TS=(RCT) | June 9th  2024 |
