# Supplementary material for: Effects of nitrate supplements on cardiopulmonary fitness at high altitude: A meta-analysis of nine randomized controlled trials
Source: PLoS One. 2025 Apr 9;20(4):e0319667. doi: 10.1371/journal.pone.0319667 (PMC11981189; doi:10.1371/journal.pone.0319667)
Supplement: S3 Table — (DOCX) [file pone.0319667.s003.docx]

Supplementary table 3: ROB 2 tool for included RCT.

| **Study ID** | **Domain 1** | **Domain 2** | **Domain 3** | **Domain 4** | **Domain 5** | **Domain 6** | **Domain 7** |
| --- | --- | --- | --- | --- | --- | --- | --- |
| Arnold, 2015 | Low | Low | Low | Low | Low | Low | Low |
| Hennis, 2016 | Low | Low | High | Low | Low | Low | Low |
| Shannon, 2016 | Low | Low | Low | Low | Low | Low | Low |
| Rossetti, 2017 | Low | Low | Low | Low | Low | Low | Low |
| Shannon, 2017 | Low | Low | Unclear | Unclear | Low | Low | Low |
| Kent, 2019 | Unclear | Unclear | Low | Unclear | Low | Low | Low |
| Robinson, 2020 | Low | Unclear | Low | Unclear | Low | Low | Low |
| Marshall 2021 | Low | Low | High | Unclear | Low | Low | Low |
| Hennis, 2022 | Low | High | Low | Low | Unclear | Low | Low |

Note: domain l-7 in heading signified: Domain 1: Random sequence generation(selection bias); Domain 2:Allocation concealment (selection bias); Domain 3:Blinding of participants and personnel(performance bias);Domian4:Blinding of outcome assessment(detection bias); Domain 5:Incomplete outcome data (attrition bias);Domain 6:Selective reporting (reporting bias); Domain 7:Other bias
